# Supplementary material for: 500 microkelvin nanoelectronics
Source: Nat Commun. 2020 Mar 20;11:1492. doi: 10.1038/s41467-020-15201-3 (PMC7083907; doi:10.1038/s41467-020-15201-3)
Supplement: Supplementary file 1 — Supplementary Information [file 41467_2020_15201_MOESM1_ESM.pdf]

# Supplementary Information

# 500 microkelvin nanoelectronics

Matthew Sarsby,[1,*] Nikolai Yurttagül,[1,*] and Attila Geresdi[1,†]

[1] *QuTech and Kavli Institute of Nanoscience,*
*Delft University of Technology, 2600 GA Delft, The Netherlands*

* These authors contributed equally to this work.

† Present address: Department of Microtechnology and Nanoscience, Chalmers University of Technology, SE 41296 Gothenburg, Sweden; e-mail: geresdi@chalmers.se

## SUPPLEMENTARY FIGURES

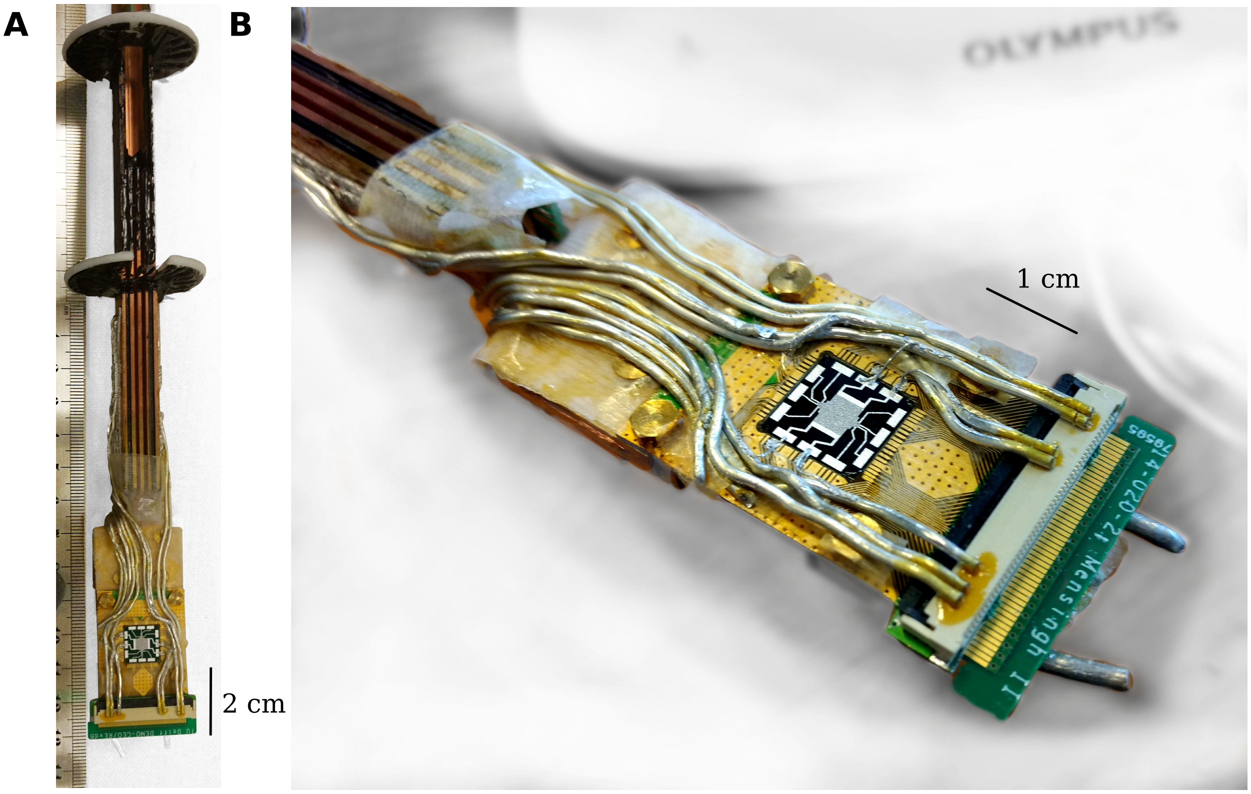


Supplementary Figure 1. The mechanical design of the cooling stage. **(A)** The lower section of the nuclear cooling stage, with a cm/mm ruler for scale. **(B)** Zoomed in view of the of the the wiring around the chip. Two indium wires come off each nuclear stage, and bonding with small indium wire by hand makes the final connection to the chip.

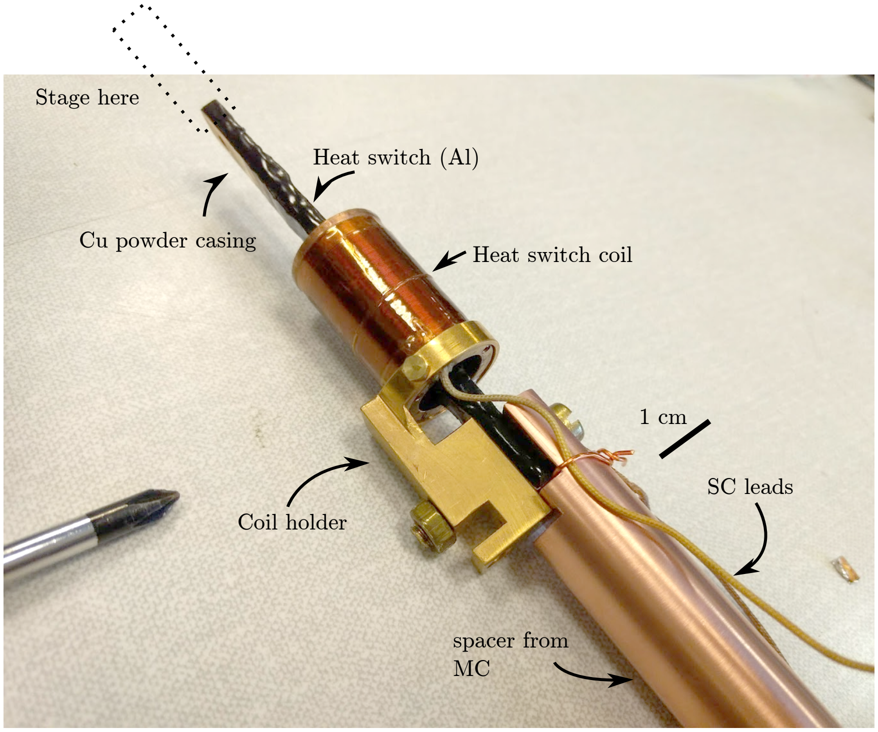


Supplementary Figure 2. The aluminium and copper foil heat switch and the switching solenoid.

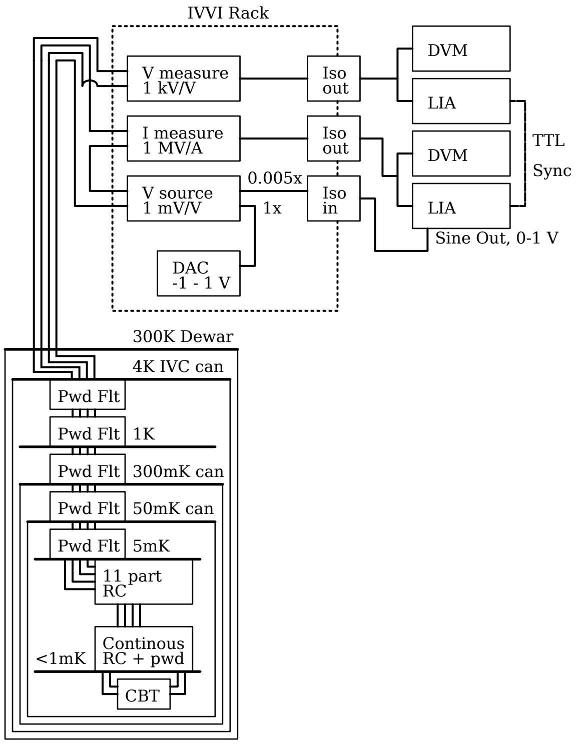


Supplementary Figure 3. A functional block diagram and filtering of the electronic measurement circuit.

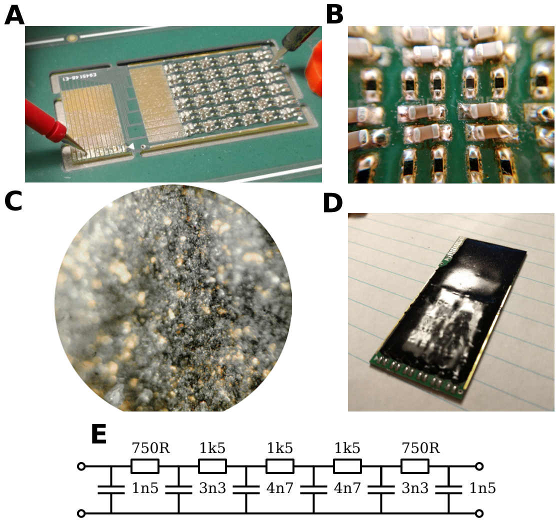


Supplementary Figure 4. The millikelvin filtering board. **(A)** The combination of meandering traces and 11 component RC filters for 12 lines per board. **(B)** The capacitor solder pad sits directly on top of a via to the ground plane for low electronic series resistance and low trace inductance. **(C)** Optical micrograph of the copper epoxy cross section. Each grain is $10 - 30\,\mu$m diameter with an approximate $50\,\mu$m spacing between grains. **(D)** Covering the board and traces with epoxy with copper powder. **(E)** The electronic equivalent circuit for a single line.

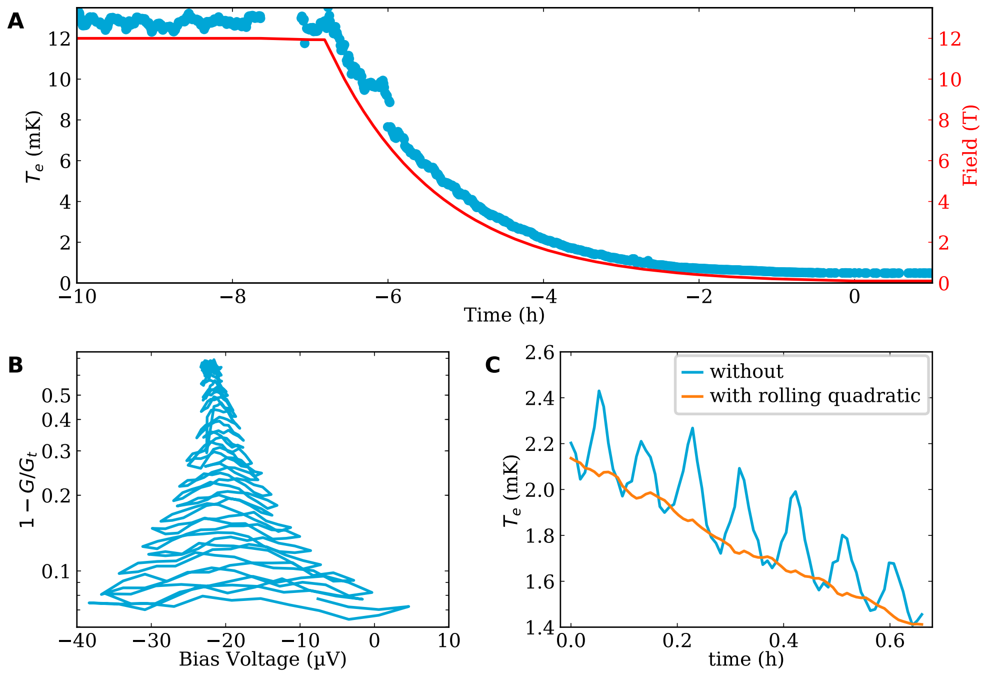


Supplementary Figure 5. Tracking of the zero bias conductance to mitigate bias drifts and thermo-voltages. **(A)** The magnetic field (solid red line) and electron temperature (cyan dots) follow the exponential ramp. **(B)** The measured conductance and bias voltage tracking during the experiment. **(C)** The raw electron temperature based on the measured conductance (cyan line) demonstrates the sensitivity of $T_e$ to small offset voltages. Orange line follows the minima by a rolling quadratic fit.

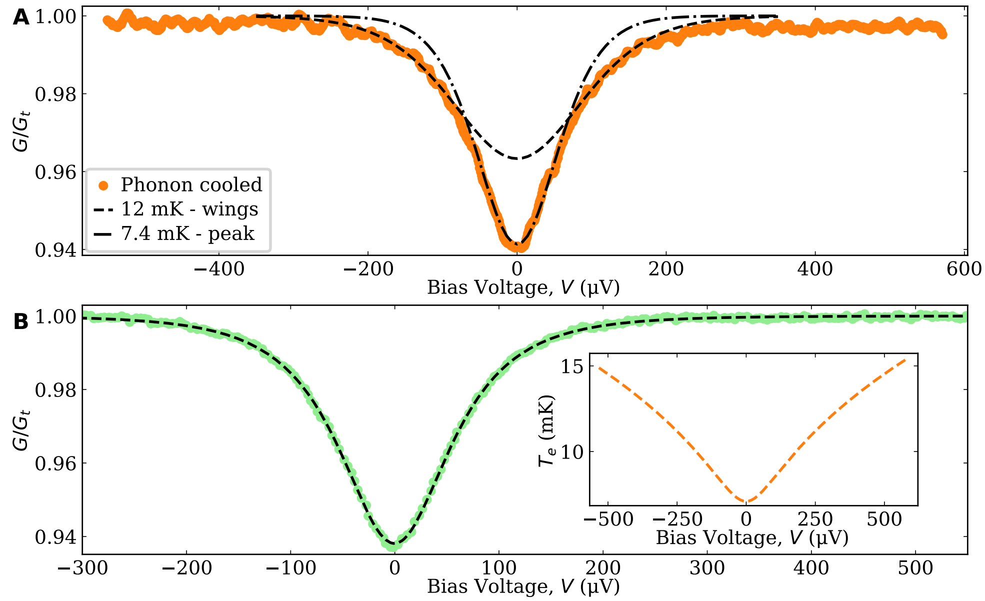


Supplementary Figure 6. **(A)** Experimental data (orange circles) with the best fit near zero bias (dashed-dotted line) and on the side of the conductance dip (dashed line) yielding different electron temperatures. **(B)** Using the overheating model, the entire measured trace (green circles) matches the fit (dashed line) with $T_{\mathrm{p}} = 7.00 \pm 0.03\,\mathrm{mK}$, $\dot{Q}_{\mathrm{par}} = 6.4 \pm 0.2\,\mathrm{aW}$ and $\Sigma = (4 \pm 1) \times 10^{7}\mathrm{Wm}^{-3}\mathrm{K}^{-5}$. The inset shows the voltage bias-dependent electron temperature.

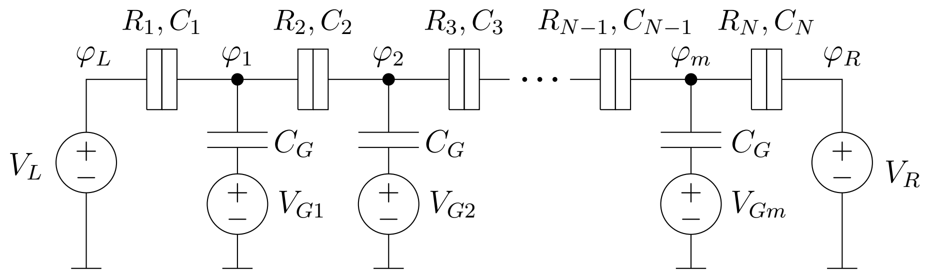


Supplementary Figure 7. The equivalent circuit of a single tunnel junction chain in the CBT.

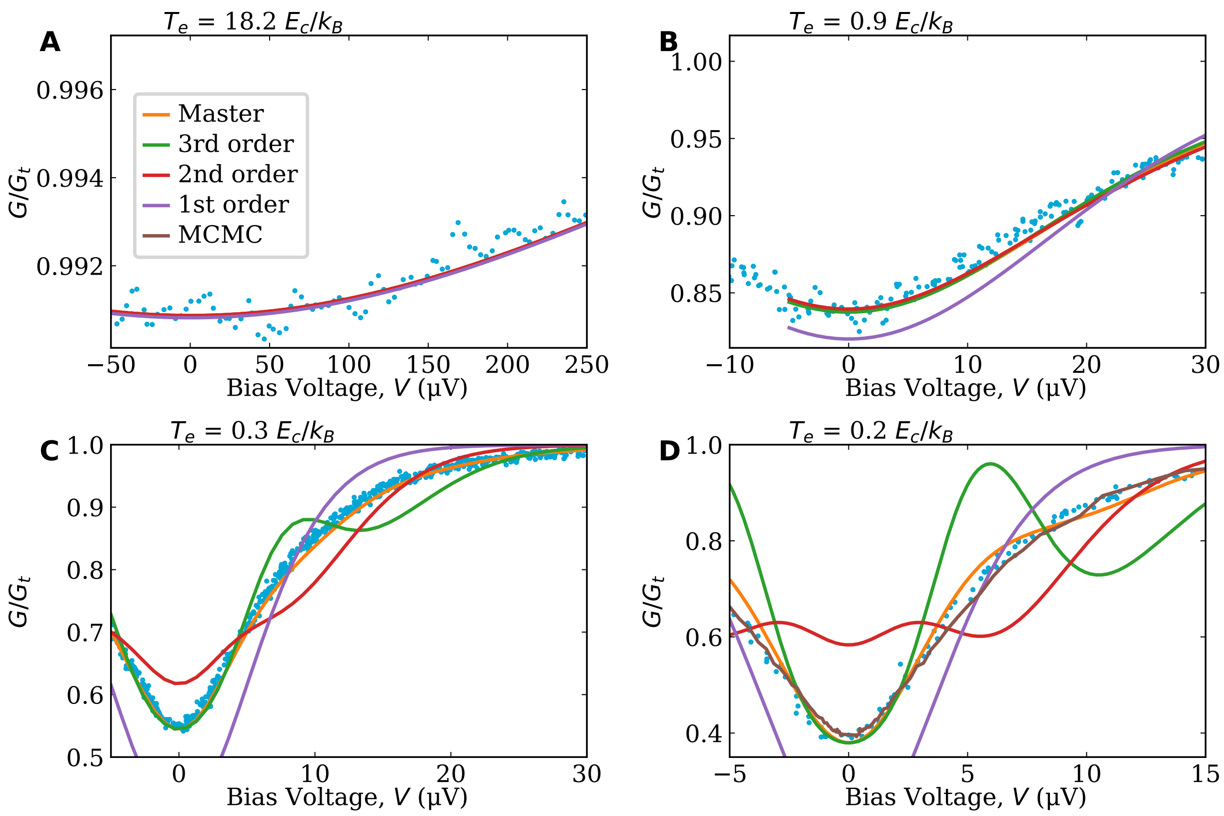


Supplementary Figure 8. Comparison of the CBT conductance models at different temperatures. The experimental data is represented by cyan circles in all panels. The following numerical models are shown as solid lines (see common legend in panel A): the full master equation model in the universal regime, 1st, 2nd and 3rd order terms in $u_{\mathrm{n}}$ in Eq. (S2), and the Markov chain Monte Carlo model, averaged over random charge configurations. Note that all models describe the data at high temperatures ($T_{\mathrm{e}} \ll E_{\mathrm{C}}/k_{\mathrm{B}}$), whereas in the low temperature regime ($T_{\mathrm{e}} \sim E_{\mathrm{C}}/k_{\mathrm{B}}$), the low order approximations fail to account for the measurement. At the lowest temperature in this study ($T_{\mathrm{e}} = 0.2E_{\mathrm{C}}/k_{\mathrm{B}}$), only the statistical Markov chain Monte Carlo model fits the experimental curve.

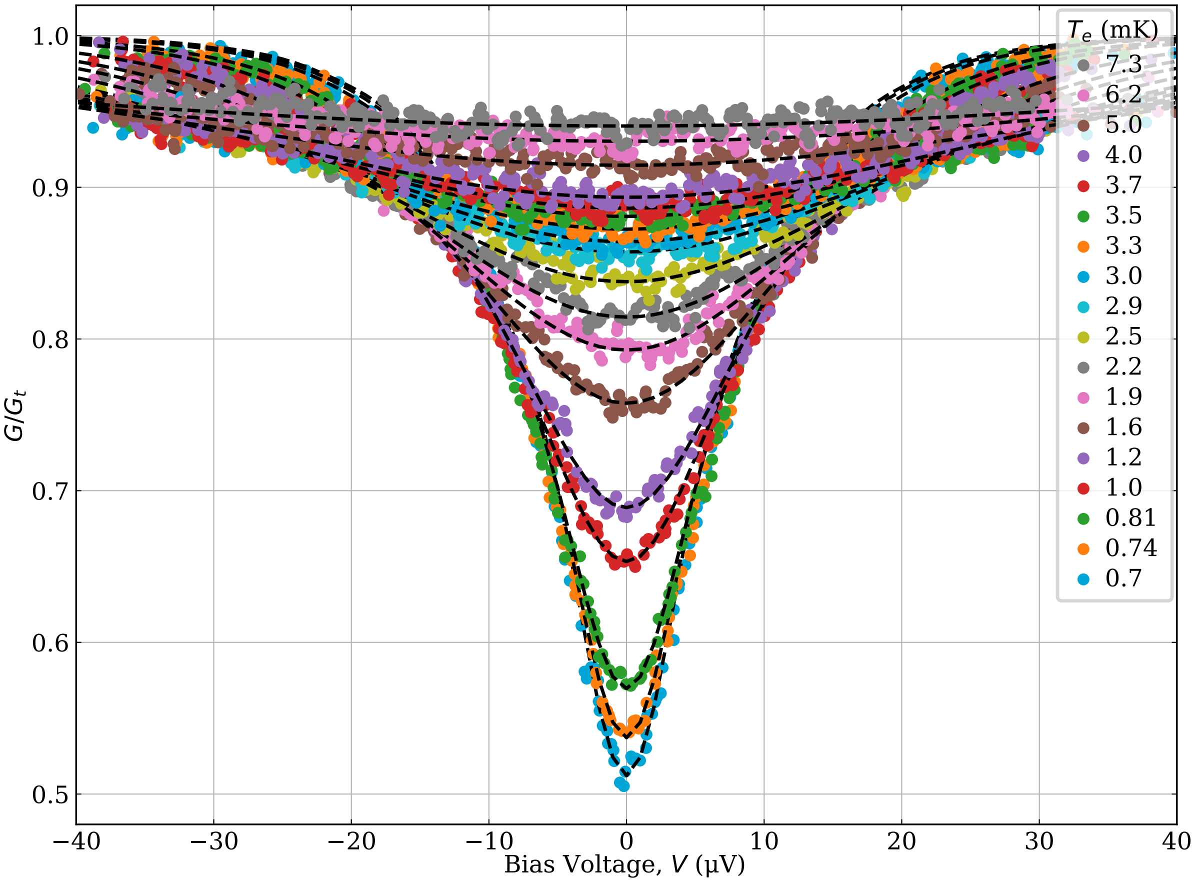


Supplementary Figure 9. Finite bias charging curves of the CBT from the microkelvin to the millikelvin regime, the fitted electron temperatures are shown in the legend. The dashed lines are the fitted theoretical curves based on the master-equation model for $T_\mathrm{e} > 1\,\mathrm{mK}$ and on the charge-offset averaged MCMC model for lower temperatures.

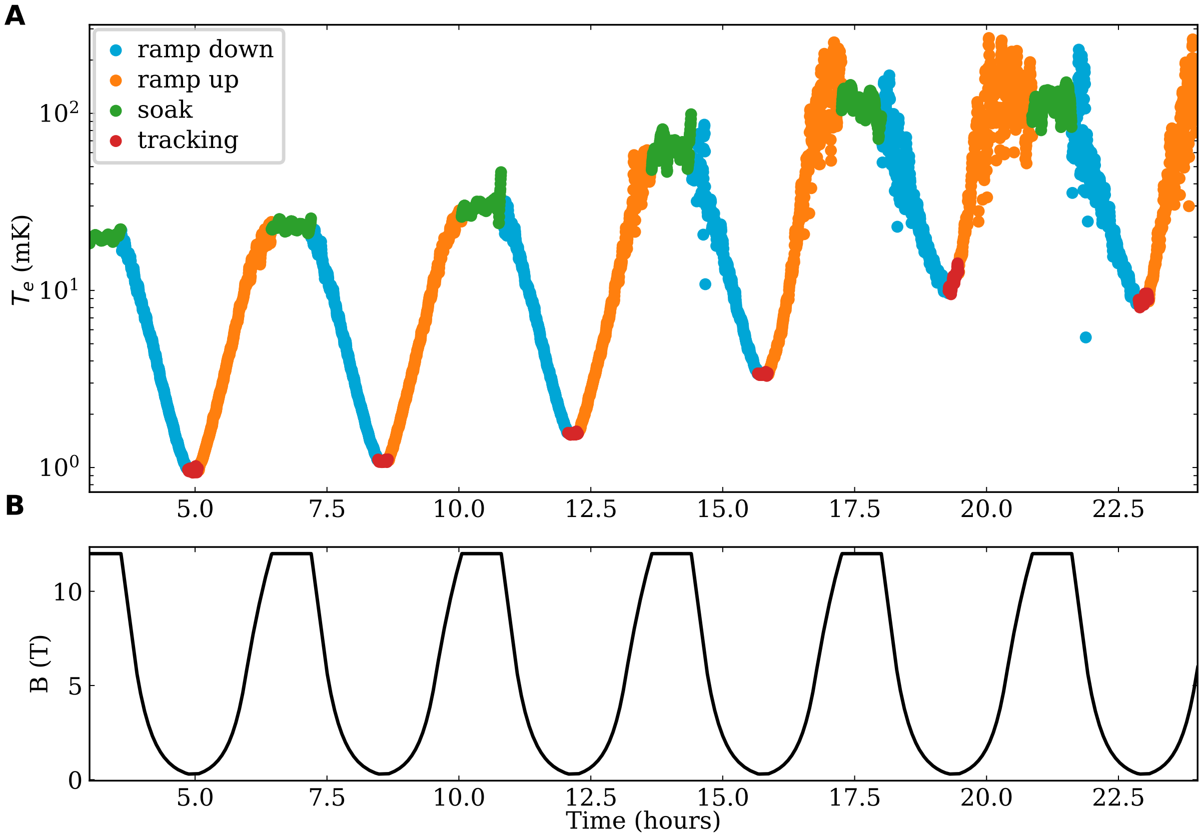


Supplementary Figure 10. Raw datasets for measuring the internal magnetic field of indium.

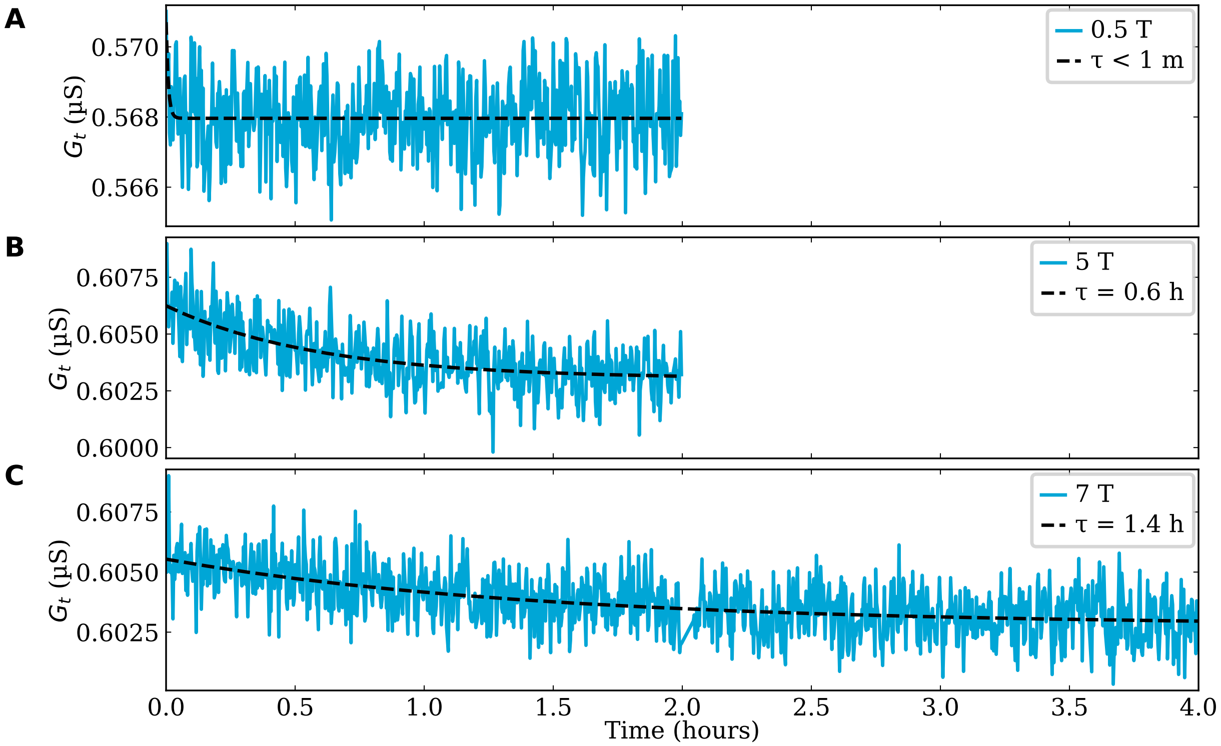


Supplementary Figure 11. Thermal relaxation times in the phonon-cooled regime at different magnetic fields when connected to the mixing chamber heated to 28 mK. The heat switch was closed when taking these datasets.

## SUPPLEMENTARY NOTE 1: THE CBT FIT PROCEDURE

### The high temperature, universal regime

A CBT is an array of tunnel junctions that enclose mesoscopic islands of finite charging energy. The tunnelling of electrons over the device is suppressed by Coulomb blockade. The temperature is acquired by measuring a characteristic dip in differential conductance, $G(V)$ below the background $G_{\mathrm{t}}$, around zero bias. If the change in conductance is small, at the high end of the working range, then the width of the conductance dip is a primary measure of the electron temperature, $eV_{1/2} = 5.439Nk_{\mathrm{B}}T/e$. The depth of the dip depends on the charging energy of the device, which is set during fabrication by the capacitance of the islands, $C_{\Sigma}$, together with the number of junctions, $N$, yielding $E_{\mathrm{C}} = (N-1)/N \cdot e^2/C_{\Sigma}$.

There are high temperature approximations for the full shape of the charging curve[1]. For a CBT working in the intermediate temperature range, above $E_{\mathrm{C}}/k_{\mathrm{B}}T > 0.4$, the charging curve can be well described with a third order approximation [2].

$$G/G_{\mathrm{t}} = 1 - u_{\mathrm{n}} g(v_{\mathrm{n}}) - \tfrac{1}{4}u_{\mathrm{n}}^2\Big(g''(v_{\mathrm{n}})h(v_{\mathrm{n}}) + g'(v_{\mathrm{n}})h'(v_{\mathrm{n}})\Big) - \tfrac{1}{8}u_{\mathrm{n}}^3\Big(\tfrac{1}{4}g''''(v_{\mathrm{n}})h(v_{\mathrm{n}})^2 + \tfrac{1}{3}g''(v_{\mathrm{n}}) + \tfrac{1}{2}g'''(v_{\mathrm{n}})h'(v_{\mathrm{n}})h(v_{\mathrm{n}})\Big)\ldots \quad (1)$$

Here,

$$h(x) = x\coth(x/2), \quad (2)$$

$$u_{\mathrm{n}} = E_{\mathrm{C}}/(k_{\mathrm{B}}T), \quad (3)$$

$$v_{\mathrm{n}} = eV/(Nk_{\mathrm{B}}T), \quad (4)$$

$$g(x) = \big(x\sinh(x) - 4\sinh^2(x/2)\big)\,/\,\big(8\sinh^4(x/2)\big)\,. \quad (5)$$

The derivative functions, such as $g''''$, are calculated symbolically with sympy (`www.sympy.org`) and converted to Python functions. These can not be evaluated numerically at zero bias, however in the limit of $v_{\mathrm{n}} \to 0$, equation 1 can be written as

$$\Delta G/G_{\mathrm{t}} = u_{\mathrm{n}}/6 - u_{\mathrm{n}}^2/60 + u_{\mathrm{n}}^2/630 + \ldots, \quad (6)$$

where $\Delta G = G_{\mathrm{t}} - G$. We note that the zero bias quantities are numerically recovered at $|v_{\mathrm{n}}| \lesssim 0.005$.

Measurements of a set of charging curves at high temperatures, $E_\mathrm{C} \ll k_\mathrm{B}T$, are used to calibrate the charging energy and the background conductance of the device. Once self-calibrated in this way, the zero bias conductance yields the electron temperature. We always numerically invert $G(T_\mathrm{e})$ to get $T_\mathrm{e}(G)$ used for thermometry.

### Fit of overheated CBT charging curves

We observe that the conductance curves of the phonon-cooled CBT are distorted in the low temperature regime, $T_\mathrm{e} \lesssim 10\,\mathrm{mK}$. This effect is attributed to the Joule heating of the device at finite voltage biases in combination with weak electron-phonon coupling [3]:

$$T_\mathrm{e} = \sqrt[5]{\frac{(V/N)^2 + R_\mathrm{t}\dot{Q}_\mathrm{par}}{R_\mathrm{t}\Sigma\Omega} + T_\mathrm{p}^5} \tag{7}$$

The temperature in equation 1 or in the single electron tunnelling model of the master equations, is modified by equation 7. The fitting parameters are now $T_\mathrm{p}$, $\dot{Q}_\mathrm{par}$ and $\Sigma$ rather than a constant $T_\mathrm{e}$. The resulting standard deviations from the least square fitting are processed to calculate the uncertainty of $T_\mathrm{e}$:

$$\sigma_{T_\mathrm{e}} \approx \frac{T_\mathrm{e}}{5}\left(\frac{5\sigma_{T_\mathrm{p}}}{T_\mathrm{p}} + \sqrt{\left(\frac{\sigma_{\dot{Q}_\mathrm{par}}}{\dot{Q}_\mathrm{par}}\right)^2 + \left(\frac{\sigma_\Sigma}{\Sigma}\right)^2}\right). \tag{8}$$

### The Markov chain Monte Carlo model of the CBT

At low temperatures ($E_\mathrm{C} \gtrsim k_\mathrm{B}T$), the CBT conductance is sensitive to uncontrolled offset charges in the array [2]. In this regime, we statistically analyse the conductance of the entire device using the Markov chain Monte Carlo approach and calculate the average and standard deviation of the conductance for a given $T_\mathrm{e}$.

A circuit model of the device is shown in Supplementary Figure 7. We assume that array is homogeneous, $R_i = R$ and $C_i = C_0$. $C_\mathrm{G}$ is the capacitance to ground and for our device $C_\mathrm{G} \ll C_0$. We constrain the offset charge in the interval $[-e/2, e/2]$. Any background charge outside of this interval is compensated by single electron tunnelling.

The voltage over the device is $V_0 = \varphi_\mathrm{L} + \varphi_\mathrm{R}$, where $\varphi_\mathrm{L} = V_0/2$ and $\varphi_\mathrm{R} = -V_0/2$ sets a symmetric bias. The potential difference, $V_i$, over each of the junctions depends on the

complete charge state on all of the islands. Calculating $V_i$ and $\varphi_i$ is done by a matrix equation relating the charges on the islands to the island potentials $\varphi_i$: [4].

$$\varphi_{i-1} + \left(2 + \frac{C_\mathrm{G}}{C_0}\right)\varphi_i + \varphi_{i+1} = \frac{Q_i}{C_0}. \tag{9}$$

Here, $Q_i$ is the sum of the charge which is induced on the island by excess charge carriers, the gate charge and the background charge. From a given charge distribution $\{Q_i\}_p$, the system can transition into $2N$ different charge configurations due to forward or reverse tunnelling over any of $N$ junctions, $\{Q_i\}_{p+1}$ . The index $p$ denotes a specific step of a Markov chain. Electrons tunnel over the $i^{\text{th}}$ junction with a rate $\Gamma_i^{\pm}$[5]. The indexed sign describes the tunnelling direction:

$$\Gamma_i^{\pm} = \frac{1}{e^2 R_i}\frac{\Delta F_i^{\pm}}{1 - \exp(-\Delta F_i^{\pm}/k_\mathrm{B}T)}. \tag{10}$$

The change in free energy, $\Delta F$, describes a single electron tunnelling event from island $i$ to island $i \pm 1$ and is equal to the electrostatic potential difference between the islands before, $\varphi$, and after, $\varphi'$, tunnelling[4]:

$$\Delta F^{\pm} = \frac{e}{2}\left(\varphi_{i\pm1} + \varphi'_{i\pm1}\right) - \frac{e}{2}\left(\varphi_i + \varphi'_i\right). \tag{11}$$

The current through the device from step $p$ to $p+1$ is the ratio of the charge moving out of this state, $\Delta Q_p$ and the expectation lifetime of the charge state, $\Delta t_p$. Both terms can be expressed using the tunnelling rates $\Gamma_i^{\pm}$ [4]. For a Markov chain with $n$ steps the total simulation time is calculated as

$$\Delta t = \sum_{p=1}^{n} \Delta t_p = \sum_{p=1}^{n}\left[\sum_{i=1}^{N}\left(\frac{1}{\Gamma_i^{+} + \Gamma_i^{-}}\right)\right]_p, \tag{12}$$

$$\Delta Q = \sum_{p=1}^{N} \Delta Q_p = \sum_{p=1}^{n}\left[\frac{e}{N}\frac{\sum_i\left(\Gamma_i^{+} - \Gamma_i^{-}\right)}{\sum_i\left(\Gamma_i^{+} + \Gamma_i^{-}\right)}\right]_p. \tag{13}$$

In thermal equilibrium, the current through each junction is equal $I = I_1 = I_2 = ... = I_N$. The steady state current of a instance of the Markov chain is $I = \Delta Q/\Delta t$.

The differential conductance is evaluated with a small bias window. We use a Monte Carlo approach and seed the system a random offset charge between $[-\frac{e}{2}, \frac{e}{2}]$ on each island. Repeating the Monte Carlo sampling of $G$ with a given $\{Q_i\}$ yields the tunnel conductance distribution $\{G\}$ of a single one-dimensional array of tunnel junctions at the given temperature. To account for $M$ parallel chains, we randomly sample $M$ elements from $\{G\}$ and compute the summed up conductance.

## SUPPLEMENTARY REFERENCES


[1] Farhangfar, S., Hirvi, K., Kauppinen, J., Pekola, J. & Toppari, J. One dimensional arrays and solitary tunnel junctions in the weak Coulomb blockade regime: CBT thermometry. *J. Low Temp. Phys.* **108**, 191–215 (1997).

[2] Feshchenko, A. *et al.* Primary thermometry in the intermediate Coulomb blockade regime. *J. Low Temp. Phys.* **173**, 36–44 (2013).

[3] Kautz, R. L., Zimmerli, G. & Martinis, J. M. Self-heating in the Coulomb-blockade electrometer. *J. Appl. Phys.* **73**, 2386–2396 (1993).

[4] Hirvi, K., Paalanen, M. & Pekola, J. Numerical investigation of one-dimensional tunnel junction arrays at temperatures above the Coulomb blockade regime. *J. Appl. Phys.* **80**, 256–263 (1996).

[5] Pekola, J. P., Hirvi, K. P., Kauppinen, J. P. & Paalanen, M. A. Thermometry by arrays of tunnel junctions. *Phys. Rev. Lett.* **73**, 2903–2906 (1994).
